# Vaginal, sexual and urinary symptoms following hysterectomy: a multi-centre randomized controlled trial

**DOI:** 10.1186/s40695-020-0049-2

**Published:** 2020-03-02

**Authors:** Chanil Ekanayake, Arunasalam Pathmeswaran, Rasika Herath, Prasantha Wijesinghe

**Affiliations:** 1 Department of Clinical Sciences, Faculty of Medicine, General Sir John Kotelawala Defence University, Ratmalana, Sri Lanka; 2 Department of Public Health, Faculty of Medicine, University of Kelaniya, Kelaniya, Sri Lanka; 3 Department of Obstetrics & Gynaecology, Faculty of Medicine, University of Kelaniya, Kelaniya, Sri Lanka

**Keywords:** Non-descent vaginal hysterectomy, Vaginal symptoms, Sexual symptoms, Urinary symptoms, Randomized controlled trial, Total abdominal hysterectomy, Total laparoscopic hysterectomy

## Abstract

**Background:**

Hysterectomy is the most common major gynaecological procedure. The aim of this study was to study vaginal, sexual and urinary symptoms following total abdominal hysterectomy (TAH), non-descent vaginal hysterectomy (NDVH) and total laparoscopic hysterectomy (TLH) in a low resource setting.

**Methods:**

A multi-centre randomized controlled trial (RCT) was conducted in two public sector hospitals in Sri Lanka. Participants were patients requiring hysterectomy for non-malignant uterine causes. Exclusion criteria were uterus> 14 weeks, previous pelvic surgery, medical illnesses which contraindicated laparoscopic surgery, and those requiring incontinence surgery or pelvic floor surgery.

Vaginal, sexual function and urinary symptoms were assessed by the validated translations of ICIQ-VS and ICIQ-FLUTS questionnaires. Post-operative improvement (pre-operative – post-operative) was assessed.

**Results:**

There was an improvement (median (IQ1-IQ3) in vaginal symptoms [TAH 6(2–8) vs 4(0–8), *p* < 0.001; NDVH 6(4–8.5) vs 5(0–8), p < 0.001; TLH 4(2–10.5) vs 4(0–10), p < 0.001], urinary flow symptoms [TAH 2(1–4) vs 1 (0–3), p < 0.001; NDVH 3 (2–5) vs 2 (0.5–4), p < 0.001; TLH 1(1–4) vs 1(0–3), *p* < 0.05], urinary voiding symptoms [TAH 0(0–0) vs 0(0–0), *p* = 0.20; NDVH 0(0–1) vs 0(0–0.8), *p* < 0.05; TLH 0(0–0) vs 0(0–0), p < 0.05] and urinary incontinence symptoms [TAH 0(0–2) vs 0(0–2), *p* = 0.06; NDVH 0(0–3) vs 0(0–3), *p* < 0.001; TLH 0(0–3) vs 0(0–2), *p* < 0.05] at 1-year (TAH *n* = 47, NDVH *n* = 45, TLH n = 47). There was an improvement in sexual symptoms only in the TLH group [TAH 0(0–11.5) vs 0(0–14), *p* = 0.08); NDVH 0(0–0) vs 0(0–0), *p* = 0.46; TLH 0(0–0) vs 0(0–4), p < 0.05].

There was no significant difference among the three different routes in terms of vaginal symptoms score [TAH 2 (0–2), NDVH 0 (0–2), TLH 0 (0–2), *p* = 0.33], sexual symptoms [TAH 0 (0–0), NDVH 0 (0–0), TLH 0 (0–0), *p* = 0.52], urinary flow symptoms [TAH 0 (0–1), NDVH 0 (0–1), TLH 0 (0–2), *p* = 0.56], urinary voiding symptoms [TAH 0 (0–0), NDVH 0 (0–0), TLH 0 (0–0), *p* = 0.64] and urinary incontinence symptoms [TAH 0 (0–0), NDVH 0 (0–1), TLH 0 (0–1), *p* = 0.35] at 1-year.

**Conclusions:**

There was a post-operative improvement in vaginal symptoms and urinary symptoms in all three groups. There was no significant difference in pelvic organ symptoms between the three routes; TAH, NDVH and TLH.

**Trial registration:**

Sri Lanka clinical trials registry, SLCTR/2016/020 and the International Clinical Trials Registry Platform, U1111–1194-8422, on 26 July 2016. Available from: http://slctr.lk/trials/515

## Background

Hysterectomy is the most commonly performed major gynaecological operation, with up to 100,000 procedures performed annually in the United Kingdom [1, 2]. During hysterectomy, the surgical dissection disrupts the normal anatomy and local nerve supply. Therefore it would seem logical to hypothesize that pelvic organ function might be adversely affected [3]. Pelvic organ function is a long-term outcome measure following hysterectomy that should be evaluated in terms of vaginal, sexual and urinary function and considered as an indicator when deciding the optimum route of hysterectomy [4–6].

There is a knowledge gap in terms of post-hysterectomy pelvic organ function comparing the three main routes; total abdominal hysterectomy (TAH), non-descent vaginal hysterectomy (NDVH) and total laparoscopic hysterectomy (TLH) [7]. Furthermore most prospective studies show an improvement in female lower urinary tract symptoms following hysterectomy [8, 9]. However, there are no published studies on pelvic organ function following hysterectomy in Sri Lanka.

Therefore, our objective was to assess vaginal, sexual, and urinary symptoms using the international consultation on incontinence modular questionnaire on vaginal symptoms (ICIQ-VS) and the international consultation on incontinence modular questionnaire on female lower urinary tract symptoms (ICIQ-FLUTS) questionnaires in women undergoing TAH, NDVH and TLH.

## Methods

### Design, setting and participants

A pragmatic multi-centre three arm (parallel groups) randomized controlled trial (RCT) was designed in accordance to Consolidated Standards of Reporting Trials (CONSORT) recommendation for pragmatic trials (Additional file 1) [10]. The study was conducted in the professorial gynaecology unit of the North Colombo Teaching Hospital-Ragama, Sri Lanka and the gynaecology unit of the District General Hospital-Mannar, Sri Lanka from 1st August 2016 to 31st October 2018. Eligible participants were patients requiring hysterectomy for non-malignant uterine causes. Exclusion criteria were uterus> 14 weeks, previous pelvic surgery, those requiring incontinence surgery or pelvic floor surgery, and any medical illness which caution/contraindicate laparoscopic surgery. Eligible patients were aware that they would be randomly assigned to undergo one of the three procedures. The main exposure variables were NDVH and TLH. The control group consisted of patients undergoing TAH. Informed written consent was obtained by research assistants assigned who enrolled participants to have either a TLH, NDVH or a TAH. Patients who declined participation in the study had the standard treatment (TAH). Additional details of the protocol could be obtained from the published article on the protocol [11].

### Sample size

Sample size calculation for the trial was based on time to recover (earliest time to resume all or a combination of activities done prior to surgery; resumption of cooking, washing clothes, sexual activity and occupation) which necessitated a sample size of 49 per arm (total = 147) [11]. A retrospective analysis of power calculation at 80% with a type 1 error of 0.05, using ½ SD of the respective pelvic organ function domain (vaginal symptoms, sexual symptoms and urinary flow, voiding and incontinence symptoms) as the minimum clinically important difference at one-year follow-up, showed that a sample size of 49 per arm was adequate to assess vaginal, sexual and urinary symptoms [12–14].

### Randomization

Block randomization in multiples of three was done at each study site by opening sealed envelopes containing computer-generated block randomization numbers, with block sizes of six and nine to ensure roughly equal numbers of patients in each arm at any point in the study. The patients and medical team were not blinded to the intervention.

### Outcome measurements

Urinary and sexual function were assessed by the validated Sinhala and Tamil translations of ICIQ-FLUTS and ICIQ-VS which were obtained from the International Consultation on Incontinence (ICI) [15–17]. Bowel symptoms were assessed by a questionnaire based on a study done by Tharkar et al [18]. These questionnaires were used pre-operatively, six months and up to one year post-hysterectomy to detect changes in pelvic organ function. The questionnaires were administered by trained data collectors.

### Data analysis

The data analysis was by intention-to-treat. Data were checked for normality and non-parametric data were described using medians and interquartile range. Kruskal-Wallis test was used to check for differences among the three groups. Wilcoxon-rank test was used to compare the pre-operative score to post-operative score to assess for improvement following surgery.

## Results

**Fig. 1:**
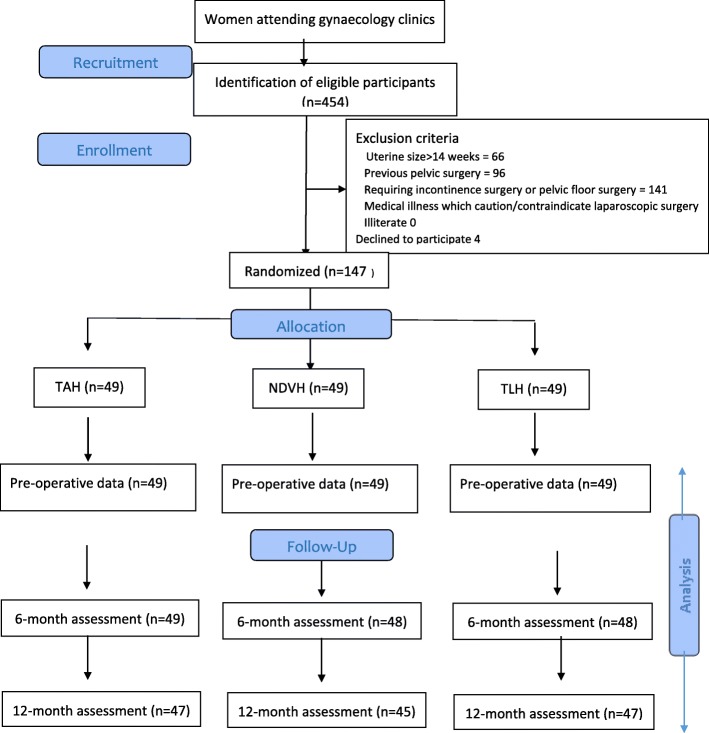
Participant flow diagram

The participant flow diagram is shown in Fig. 1. Out of the 147 patients, 71 (48.3%) (TAH-24, NDVH-23 and TLH-24) were from Mannar whilst 76 (51.7%) (TAH-25, NDVH-26, and TLH-25) were from Ragama. Over one year of follow-up, 139 of the 147 patients (94.6%)completed the entire follow-up. In the TAH arm, two patients from Mannar were lost to follow-up after 6-months as they moved to a different location. In the TLH arm, two patients from Mannar (one after 6-weeks and another after 6-months) were lost to follow-up for similar reasons. In the NDVH arm, two patients from Mannar were lost to follow-up after 3-months and 6-months respectively, whilst from Ragama, one patient died following a myocardial infarction after 6-months and another was followed up at a different hospital from 6-months onwards.

Basic characteristics of the study population in each treatment arm were similar in terms of age, BMI and parity (Table 1). The three groups did not differ pre-operatively in terms of vaginal symptoms score [TAH 6(2–8), NDVH 6 (4–8.5), TLH 4 (2–10.5), *p* = 0.63], sexual symptoms [TAH 0 (0–11.5), NDVH 0 (0–0), TLH 0 (0–0), *p* = 0.07], urinary flow symptoms [TAH 2 (1–4), NDVH 3 (2–5), TLH 1 (1–4), *p* = 0.14], urinary voiding symptoms [TAH 0 (0–0), NDVH 0 (0–1), TLH 0 (0–0), *p* = 0.25] and urinary incontinence symptoms [TAH 0 (0–2), NDVH 0 (0–3), TLH 0 (0–3), p = 0.25] (Tables 2 and 3).

**Table 1:** Basic characteristics of study population

	TAH (*n* = 49)	NDVH (*n* = 49)	TLH (*n* = 49)	Significance (p)
**Age**	46.5	47.6	47.4	p = 0.63
[mean, (95% CI)]	(45.1–47.8)	(45.5–49.7)	(45.8–49.0)	
**BMI**	26.24	25.72	25.19	*p* = 0.51
[mean, (95% CI)]	(24.90–27.59)	(24.42–27.0)	(24.08–26.31)	
**Parity**	2.00	3.00	3.00	p = 0.20
[median, (IQ1-IQ3)]	(2.00–3.00)	(2.00–3.50)	(2.00–3.50)	
**Uterine weight** [median, (IQ1-IQ3))] (g)	124 (90–252)	111 (91–153)	141 (101–198)	*p* = 0.16

**Table 2:** Vaginal and sexual symptoms pre-operatively, 6-months and 1-year following TAH, NDVH and TLH

Pelvic organ function	TAH	NDVH	TLH	Significance
VSS pre-op [median, (IQ1-IQ3))]	6 (2–8)	6 (4–8.5)	4 (2–10.5)	p = 0.63
VSS 6-months [median (IQ1-IQ3)]	4 (0–8), p < 0.001^#^	4 (0–8), *p* < 0.001^#^	3 (0–6), *p* < 0.001^#^	
VSS 1-year [median (IQ1-IQ3)]	4 (0–8), p < 0.001^#^	5 (0–8), p < 0.001^#^	4 (0–10), p < 0.001^#^	
SSS pre-op [median, (IQ1-IQ3))]	0 (0–11.5)	0 (0–0)	0 (0–0)	p = 0.07
SSS 6-months [median (IQ1-IQ3)]	0 (0–10), *p* < 0.05^#^	0 (0–0), *p* = 0.69^#^	0 (0–0), *p* = 0.06^#^	
SSS 1-year [median (IQ1-IQ3)]	0 (0–14), *p* = 0.08^#^	0 (0–0), *p* = 0.46^#^	0 (0–4.2), p < 0.05^#^	

^#^*p* values indicate the significance of the pelvic organ function domain score for the respective route at 6-months and 1-year compared to the pre-operative score

TAH – *n* = 49 for all at 6-months except SSS (*n* = 23), *n* = 47 for all at 1-year except SSS (*n* = 22)

NDVH– *n* = 48 for all at 6-months except SSS (*n* = 29), *n* = 45 for all at 1-year except SSS (*n* = 28)

TLH– *n* = 48 for all at 6-months except SSS (*n* = 23), *n* = 47 for all at 1-year except SSS (*n* = 22)

**Table 3:** Urinary symptoms pre-operatively, 6-months and 1-year following TAH, NDVH and TLH

	TAH	NDVH	TLH	Significance
Urinary flow symptoms pre-op [median, (IQ1-IQ3)]	2 (1–4)	3 (2–5)	1 (1–4)	p = 0.14
Urinary flow symptoms 6-months [median (IQ1-IQ3)]	1 (0–3), p < 0.001^#^	2 (0–4.75), p < 0.001^#^	1 (0–3), *p* < 0.01^#^	
Urinary flow symptoms 1-year [median (IQ1-IQ3)]	1 (0–3), p < 0.001^#^	2 (0.5–4), p < 0.001^#^	1 (0–3), p < 0.05^#^	
Urinary voiding symptoms pre-op [median, (IQ1-IQ3)]	0 (0–0)	0 (0–1)	0 (0–0)	p = 0.25
Urinary voiding symptoms 6-months [median (IQ1-IQ3)]	0 (0–0), p = 0.47^#^	0 (0–0.75), p < 0.05^#^	0 (0–0), *p* = 0.47^#^	
Urinary voiding symptoms 1-year [median (IQ1-IQ3)]	0 (0–0), p = 0.20^#^	0 (0–1), p < 0.05^#^	0 (0–0), *p* < 0.05^#^	
Urinary incontinence pre-op [median, (IQ1-IQ3)]symptoms	0 (0–2)	0 (0–3)	0 (0–3)	p = 0.25
Urinary incontinence symptoms 6-months [median (IQ1-IQ3)]	0 (0–2), p = 0.06^#^	1 (0.75–3), p = 0.07^#^	0 (0–1.75), *p* = 0.21^#^	
Urinary incontinence symptoms 1-year [median (IQ1-IQ3)]	0 (0–2), p < 0.01^#^	0 (0–3), *p* < 0.01^#^	0 (0–2), p < 0.05^#^	

^#^ - *p* values indicate the significance of the pelvic organ function domain score for the respective route at 6-months and 1-year compared to the pre-operative score

TAH – n = 49 for all at 6-months and n = 47 for all at 1-year

NDVH– n = 48 for all at 6-months and n = 45 for all at 1-year

TLH– n = 48 for all at 6-months and n = 47 for all at 1-year

There was an improvement in vaginal symptoms, urinary flow symptoms, urinary voiding symptoms and urinary incontinence symptoms at 6-months and 1-year when compared with the pre-operative level for all three routes (Tables 2 and 3). There was an improvement in sexual symptoms in the TLH group at 1-year [TAH 0 (0–11.5) vs 0 (0–14), *p* = 0.08); NDVH 0 (0–0) vs 0 (0–0), *p* = 0.46; TLH 0 (0–0) vs 0 (0–4), *p* < 0.05] (Tables 2 and 3).

**Fig. 2:**
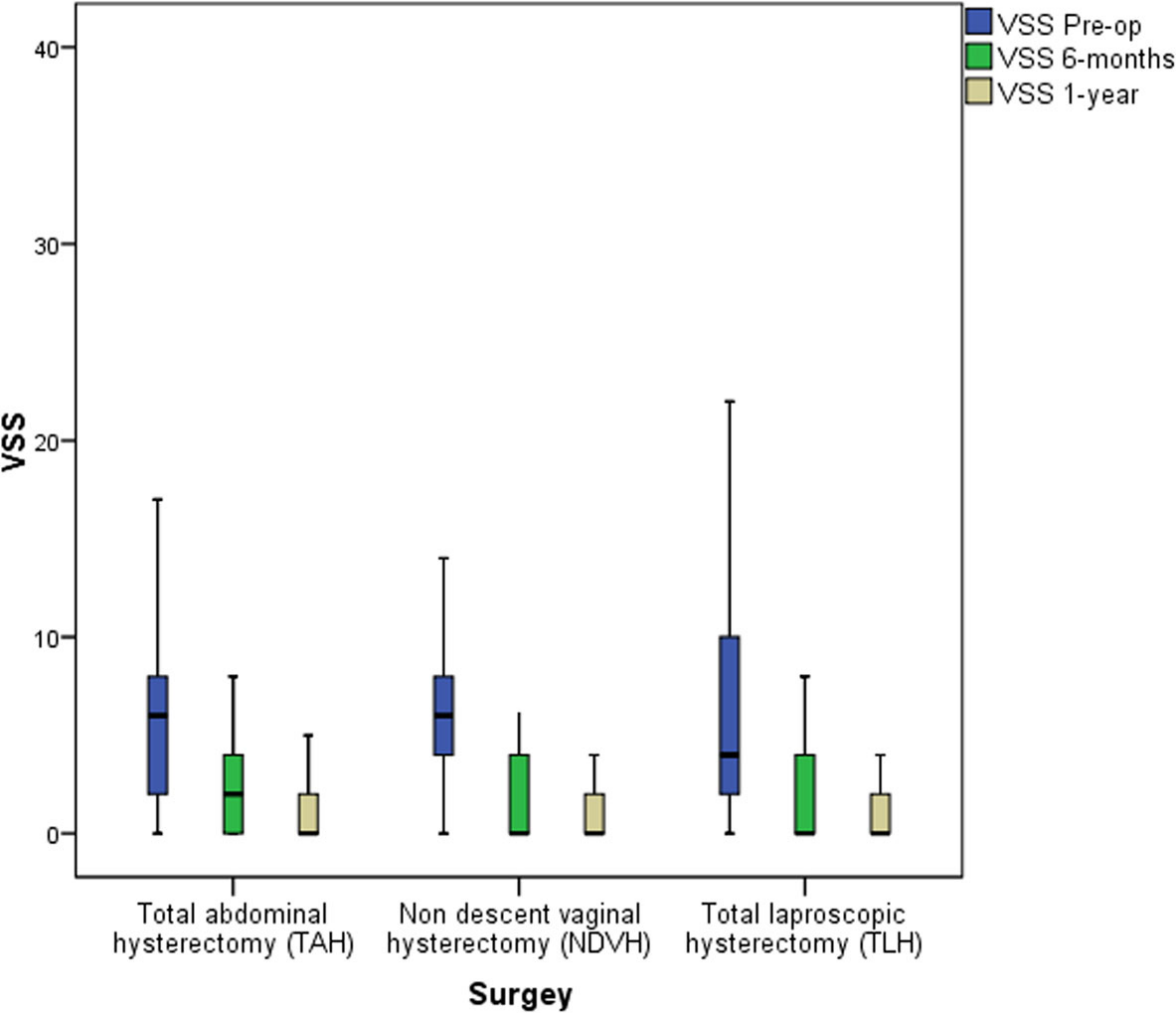
Vaginal symptoms score (VSS)

There was no significant difference among the three different routes in terms of VSS at 6-months [median (IQ1-IQ3)] [TAH 2 (0–4), NDVH 0 (0–4), TLH 0 (0–4), *p* = 0.56] and 1-year [TAH 2 (0–2), NDVH 0 (0–2), TLH 0 (0–2), *p* = 0.33] (Fig. 2).

There was no significant difference among the three different routes in terms of SSS at 6-months [median (IQ1-IQ3)] [TAH 0 (0–0), NDVH 0 (0–0), TLH 0 (0–0), *p* = 0.71] and 1-year [TAH 0 (0–0), NDVH 0 (0–0), TLH 0 (0–0), *p* = 0.52].

There was no significant difference among the three different routes in terms of urinary flow symptoms at 6-months [median (IQ1-IQ3)] [TAH 0 (0–1), NDVH 0 (0–2), TLH 0 (0–2), *p* = 0.78] and 1-year [TAH 0 (0–1), NDVH 0 (0–1), TLH 0 (0–2), p = 0.56].

There was no significant difference among the three different routes in terms of urinary voiding symptoms at 6-months [median (IQ1-IQ3)] [TAH 0 (0–0), NDVH 0 (0–0), TLH 0 (0–0), *p* = 0.45] and 1-year [TAH 0 (0–0), NDVH 0 (0–0), TLH 0 (0–0), *p* = 0.64].

There was no significant difference among the three different routes in terms of urinary incontinence symptoms at 6-months [median (IQ1-IQ3)] [TAH 0 (0–1), NDVH 0 (0–2.75), TLH 0 (0–1), *p* = 0.11] and 1-year [TAH 0 (0–0), NDVH 0 (0–1), TLH 0 (0–1), *p* = 0.35].

## Discussion

This study assessed vaginal, sexual, and urinary symptoms in Sri Lankan women undergoing TAH, NDVH and TLH. One of the major findings was that vaginal symptoms, urinary flow symptoms, urinary voiding symptoms and urinary incontinence symptoms improved at 6-months following surgery and showed further improvement at 1-year as well. This overall improvement in pelvic organ function following hysterectomy was described by Thakar [3]. The other important finding was that there was no significant difference in post-operative pelvic organ function between the three surgical approaches; TAH, NDVH and TLH which was also in keeping with available evidence [19–23].

As this was a multicentre study the involvement of multiple surgeons was inevitable and this may have raised the issue of differential expertise bias despite requiring a minimum of 25 surgeries to be a participating surgeon [24]. The issue of varying skill levels could have affected the surgical outcomes. Although the issue of multiple surgeons is a limitation to the study, it enhances the generalisability of the results to the rest of the country. Thus we chose to design a pragmatic multicentre study involving multiple surgeons for each method rather than a single centre study with a single surgeon for each method.

As this was a randomized controlled trial that was designed to detect a post-operative improvement and also to assess a difference among the three routes in terms of pelvic organ function, patients with similar symptomatology were needed. Therefore stringent exclusion criteria were adopted (uterus> 14 weeks, previous pelvic surgery, those requiring incontinence surgery or pelvic floor surgery). This would have eliminated patients with major urinary, vaginal and sexual symptoms but included women with no symptoms in several domains which resulted in a non-parametric distribution. This may have contributed to failure of the questionnaires to detect a difference among the three routes [25].

The lack of a normal distribution precluded the use of ANCOVA which would have been the ideal approach as baseline values are negatively correlated with change because patients with worse scores at baseline generally improve more than those with low scores [26]. Data transformation to logarithmic transformation or square root was not successful, and as such the difference between pre-operative scores and post-operative scores were used to assess improvement.

It was also not possible to do urodynamics to assess urinary dysfunction as it is not readily available in the Sri Lankan setting. Urodynamic studies are also not sensitive to detect subtle differences in urinary function [27]. Furthermore the association between clinical symptoms and urodynamics are poor [28]. Therefore, one may question the relevance of urodynamic testing rather than relying on patient reported outcomes by way of validated questionnaires.

Hysterectomy alone is a risk factor for early menopause even if the ovaries are conserved [29]. The impact of menopause on oestrogen deficiency and subsequent effects on female lower urinary tract symptoms (LUTS) are well documented [27]. Therefore, it is likely that menopausal status acted as a confounding factor for LUTS. However, it was not possible to assess this as serum FSH levels are not readily available in the public sector.

It is also well known that the perception of urinary voiding symptoms in women varies [27]. Furthermore women with menorrhagia and LUTS prior to surgery are likely to complain about LUTS post-hysterectomy as menorrhagia would have been resolved [27]. It is also important to note that many women have symptoms related to pelvic organs and not all post-operative complaints result from the surgery itself [27]. These are compelling reasons to interpret LUTS with caution and further strengthens the importance of documenting symptoms prior to surgery through self-reported questionnaires.

Huang and Ding found that the risk of prolapse was doubled following hysterectomy over 10-years [28]. Observational studies show that the risk of vault prolapse is higher with VH and LAVH which is probably due to the fact that these patients would have had some degree of pelvic floor weakness which would have contributed to the decision to undergo a vaginal procedure [29]. A RCT on the most common routes, abdominal, vaginal and laparoscopic would have been the best way to exclude the possible impact of the route of surgery on vault prolapse. However, this would require at least 10-years of follow-up post-hysterectomy which was not feasible in our setting.

Although 49 patients were included in each arm only 25 were sexually active to begin with with an additional 10 patients being sexually active post-hysterectomy. This increase in sexual activity may be because possible detrimental factors were eliminated following surgery [3, 30]. The fact that more women were sexually active after surgery may actually be the best indicator of a beneficial effect of hysterectomy on sexual symptoms.

Pelvic organ functions are difficult to assess due to the intimate nature of symptoms which hinders expression of the actual status of the problem. However the use of objective and validated questionnaires, for patients’ as well as for assessors has been suggested as a method to help minimize bias [10]. We believe the use of validated questionnaires overcame the problem of under-reporting in our study. Furthermore in most clinical examinations functionality is often not considered. This is especially true for pelvic organ assessments. The use of a questionnaire can address this issue and can often yield valuable information helpful to deciding upon further management of the patient.

The fact that this study was a prospective study eliminates recall bias whilst a years’ follow-up, especially in a low resource setting, has special significance where recourse to surgical management is a common due to limited availability of alternative treatments options and a lack of resources for adequate conservative management.

## Conclusions

The finding that there was a post-operative improvement in vaginal and urinary symptoms regardless of the route of hysterectomy provides valuable evidence for an under-resourced setting.

## Supplementary information

**Additional file 1.** CONSORT 2010 checklist of information to include when reporting a randomised trial*.

**Additional file 2.** Datasheet.

## Data Availability

All data generated for this article are based on the Additional file 2.

## Abbreviations

ANCOVA: ANalysis of COVAriance
CONSORT: CONsolidated Standards Of Reporting Trials
ICIQ-FLUTS: International Consultation on Incontinence Modular Questionnaire on Female Urinary Tract Symptoms
ICIQ-VS: International Consultation on Incontinence Modular Questionnaire on Vaginal Symptoms
LUTS: Lower urinary tract symptoms
NDVH: Non-Descent Vaginal Hysterectomy
PROMs: Patient reported outcome measures
RCT: Randomized controlled trial
SLCTR: Sri Lanka clinical trials registry
SSS: Sexual symptoms score
TAH: Total Abdominal Hysterectomy
TLH: Total Laparoscopic Hysterectomy
VSS: Vaginal symptoms score
